# Active Reward Processing during Human Sleep: Insights from Sleep-Related Eating Disorder

**DOI:** 10.3389/fneur.2012.00168

**Published:** 2012-11-27

**Authors:** Lampros Perogamvros, Patrick Baud, Roland Hasler, Claude Robert Cloninger, Sophie Schwartz, Stephen Perrig

**Affiliations:** ^1^Sleep Laboratory, Division of Neuropsychiatry, Department of Psychiatry, University Hospitals of GenevaGeneva, Switzerland; ^2^Department of Neuroscience, University of GenevaGeneva, Switzerland; ^3^Swiss Center for Affective Sciences, University of GenevaGeneva, Switzerland; ^4^Division of Medical Genetics and Laboratory, Department of Psychiatry, University Hospitals of GenevaGeneva, Switzerland; ^5^Department of Psychiatry, Washington University School of MedicineSt. Louis, MO, USA; ^6^Geneva Neuroscience Center, University of GenevaGeneva, Switzerland

**Keywords:** sleep, sleep-related eating disorder, reward processing, dreaming, parasomnias, mesolimbic dopaminergic system

## Abstract

In this paper, we present two carefully documented cases of patients with sleep-related eating disorder (SRED), a parasomnia which is characterized by involuntary compulsive eating during the night and whose pathophysiology is not known. Using video-polysomnography, a dream diary and psychometric examination, we found that both patients present elevated novelty seeking and increased reward sensitivity. In light of new evidence on the mesolimbic dopaminergic implication in compulsive eating disorders, our findings suggest a role of an active reward system during sleep in the manifestation of SRED.

## Introduction

Two patients consulted our Sleep Laboratory for a nighttime eating behavior. The first patient (patient A) was a 45-year-old obese (BMI 33.1) man who presented this behavior since 5 months. After falling asleep in the evening, he would sometimes find himself in the middle of the night sitting in front of the television, with a plate full of food. He also described waking up in the morning with a sweet taste in his mouth or with food on his bed, as if he had eaten some sweets during the night, especially the ones belonging to his wife. However, there was a total amnesia about these events. The patient decided to videotape these episodes and thus installed a video camera with movement-detector in his kitchen. The video recordings confirmed a nighttime eating behavior 2–3 h after sleep onset. This behavior occurred approximately once per week. The patient did not take any medication that would be likely to produce this behavior (e.g., zolpidem). Patient A had a history of sleepwalking in childhood, as well as a binge-eating disorder (BED) during the past 5 years. There was no history of other psychiatric disorder (depression, drug, or alcohol consumption).

The second patient (patient B) was a 29-year-old non-obese (BMI 25.1) man who presented a nighttime eating behavior since 3 years. He also reported waking up in the morning with a sweet taste in his mouth or with food on his bed, as if he had eaten some sweets during the night. However, there was a total amnesia about these events. Interestingly, when his wife would buy her favorite chocolate, the probability of this behavior (and the ingestion of this particular food) would increase. The patient did not take any medication that would be likely to produce this behavior (e.g., zolpidem). This patient also reported very intense dreaming, especially during the nights during which he had nighttime eating behavior. Patient B had a history of sleepwalking and sleep-talking in childhood. There was no history of other psychiatric disorder (depression, drug, or alcohol consumption).

Both patients underwent one night of videopolysomnographic evaluation. Sleep scoring was done according to the 2007 American Academy of Sleep Medicine (AASM) manual (Iber et al., 2007). Sleep scoring results are presented in Table 1.

**Table 1:** **Sleep scoring results for the two patients**.

	Patient A (45-year old)	Patient B (29-year old)
Total sleep time	7 h18	7 h20
Sleep efficiency (%)	79	94.6
N1 latency (min)	16	8
N2 latency (min)	26.3	11
REM latency	4 h33	108 min
N1 (%)	11	8
N2 (%)	66	46
N3 (%)	9	25
REM (%)	14	21.5
AHI (/h)	13.4	13.2
RERA	113	83
RDI (/h)	28.9	25.3
ODI (/h)	6.3	7.5
PLMs index (/h)	0	1
Microarousals index (/h)	27.4	28

*N1, N2, and N3, Stages 1, 2, 3 of NREM sleep; REM, rapid-eye movement sleep; AHI, apnea–hypopnea index; RERA, respiratory effort-related arousals; RDI, respiratory disturbance index; ODI, oxygen desaturation index; PLMs, periodic limb movements during sleep*.

Both patients demonstrated a mild sleep apnea syndrome [obstructive sleep apnea (OSA); Apnea–Hypopnea Index (AHI) at 13.4/h for patient A, and AHI at 13.2/h for patient B], but no nighttime eating episode. However, we noted many chewing and swallowing movements during N2 stage sleep in patient A and a dissociated arousal (persistence of slow-wave elements in the EEG in a behaviorally awaken patient) in the early morning, after a slow-wave sleep period (stage N3) in patient B. There was no paroxystical element in both wake and sleep for both patients.

Eating patterns during day and night were assessed by the Binge-eating scale (BES), a questionnaire assessing the presence of BED (Gormally et al., 1982), and the Night-Eating Questionnaire (NEQ), a questionnaire assessing the severity of night-eating syndromes (Allison et al., 2008). Both patients fulfilled the diagnostic criteria for sleep-related eating disorder (SRED; American Academy of Sleep Medicine, 2005; pp. 174 and 175), namely recurrent episodes of involuntary eating and drinking during sleep (criterion A), morning anorexia (criterion B), and absence of other sleep, medical or mental disorder, or medication/substance explaining these symptoms (criterion C). As expected, both patients had high scores on the NEQ (Allison et al., 2008), reporting unawareness of episodes, and that less than 25% of daily calories were consumed after the evening meal (Table 2). Patient A (but not patient B) also presented a BED during the day, according to the BES. This is not an uncommon finding, as daytime eating disorders have been previously associated with SRED (Winkelman et al., 2011).

**Table 2:** **Assessment of patient scores (*p*) in eating-related and psychometric questionnaires**.

	Patient A (45-year old)	Patient B (29-year old)	Normative data
Night-Eating Questionnaire (NEQ)	33	30	NEQ score > 25 “possible NES indicator”
			NEQ score > 30 “strong NES indicator”
			Allison et al. (2008)
Binge-eating scale (BES)	27	13	<17 no BED
			18–26 moderate BED
			>27 severe BED
			Gormally et al. (1982)
Temperament and Character Inventory (TCI)			Males: *n* = 195
	RD: 15.00	RD: 22.00**	RD: 14.2 (3.7)
	NS: 25.00*	NS: 26.00*	NS: 18.7 (5.3)
	NS1: 11.00**	NS1: 11.00**	NS1: 5.9 (2.3)
	P: 3.00	P: 5.00	P: 4.5 (1.9)
	HA: 1.00*	HA: 15.00	HA: 12.4 (6.0)
	S: 32.00	S: 33.00	S: 34.4 (5.9)
	C: 37.00	C: 37.00	C: 32.8 (4.8)
	ST: 21.00*	ST: 24.00*	ST: 13.2 (6.3)
			Geneva population (unpublished data)
Sensation Seeking Scale Form V (SSSV)			Males: *n* = 410
	TAS: 9	TAS: 9	TAS: 7.7 (2.2)
	ES: 8*	ES: 8*	ES: 5.0 (2.3)
	DIS: 4	DIS: 8	DIS: 6.2 (2.4)
	BS: 6*	BS: 6*	BS: 3.7 (2.0)
			Zuckerman (1994)
BIS/BAS			Males: 18–29-year old, *n* = 277
		BIS: 20	BIS: 19.3 (3.5)
		BAS reward: 19*	BAS reward: 16.8 (1.9)
		BAS fun: 14	BAS fun: 12.3 (2.2)
		BAS drive: 8	BAS drive: 10.9 (2.3)
			Males: 40–49-year old, *n* = 336
	BIS: 10*		BIS: 20 (3.7)
	BAS reward: 19*		BAS reward: 16.1 (2.1)
	BAS fun: 12		BAS fun: 10.6 (2.2)
	BAS drive: 9		BAS drive: 10.3 (2.4)
			Jorm et al. (1999)

Normative data for TCI, SSSV, and BIS/BAS scales are represented by means (μ) and SD (σ). One asterisk (*) indicates results with *z*-score = μ − *p*/σ > 1, and two asterisks (**) indicate results with *z*-score = μ − *p*/σ > 1.96. BAS, behavioral approach system; BED, binge-eating disorder; BIS, behavioral inhibition system; BS, boredom susceptibility; C, cooperativeness; DIS, disinhibition; ES, experience seeking; HA, harm avoidance; NES, night-eating syndrome; NS, novelty seeking; NS1, exploratory excitability; P, persistence; RD, reward dependence; S, self-directedness; ST, self-transcendence; TAS, thrill and adventure seeking.

In addition, we decided to assess the reward functioning and other motivational aspects of personality and behavior (e.g., approach, avoidance) in both patients. We used three psychometric questionnaires, which are widely used in both research and clinical assessments. Behavioral Inhibition System–Behavioral Approach System (BIS/BAS) is a questionnaire assessing individual differences in the sensitivity of the behavioral approach (appetitive motives) and behavioral avoidance (aversive motives) systems (Carver and White, 1994). Reward sensitivity (as measured by the BAS scale) is positively correlated to gray matter density in mesolimbic regions (ventral striatum, dorsolateral prefrontal cortex; Schweinhardt et al., 2009) and to activation of the fronto-striatal-amygdala-midbrain network, in response to images of appetizing foods (Beaver et al., 2006). Sensation Seeking Scale Form V (SSSV) is a scale assessing the personality traits of thrill and adventure seeking, disinhibition, experience seeking, and susceptibility to boredom (Zuckerman et al., 1978). Higher scores on novelty and sensation seeking in this scale (especially experience seeking) correspond to heightened mesolimbic dopaminergic (ML-DA) sensitivity (Netter et al., 1996; Hutchison et al., 1999). Finally, Temperament and Character Inventory (TCI) is an inventory for personality traits (novelty seeking, harm avoidance, persistence, and reward dependence) and characters (self-transcendence, self-directedness, and cooperativeness), which is known to correlate with specific neurobiological underpinnings (Cloninger, 1986). In particular, high exploratory excitability (a novelty seeking subscale) reflects high ML-DA activity (Leyton et al., 2002; Krebs et al., 2009), facilitated by low concentrations of D2/D3 autoreceptors in the midbrain (Zald et al., 2008). A possible association between the dopamine (DA) D4 receptor gene (DRD4) and novelty seeking has been also proposed (Ebstein and Belmaker, 1997), although the findings have been inconsistent (Lusher et al., 2001).

In Table 2 we resume the findings of psychometric assessment in the two patients. Apart from individual differences (Patient A showed low harm avoidance and behavioral inhibition scores and patient B demonstrated a particularly elevated reward dependence score), both patients had elevated novelty seeking [with the exploratory excitability subscale (NS1) being particularly increased] and self-transcendence on the TCI, increased experience seeking and boredom susceptibility in the SSSV, and reward responsiveness in the BIS/BAS scale, compared to population means (Table 2). Taken together, these findings suggest increased reward sensitivity and risk taking.

Finally, a dream diary was kept by both patients during 2 weeks after the polysomnography. Over the 2 weeks of the dream diary, patient A wrote down two detailed dream reports, which were both characterized by false recognition, increased self-confidence, and curiosity toward novel stimuli (especially unknown persons). Interestingly, these dreams occurred during the same nights when he also had nighttime eating behavior at home. Over the 2 weeks of the dream diary patient B reported also two dreams. In one dream, the patient was a prisoner in a dangerous place in Southern Europe and was trying to escape. In another dream, the patient had to cross several uninhabitable mountains facing dangerous and unknown situations (wild animals, parachuting); feelings of fear, and excitement were prominent in these dreams. Again, and like for patient A, these dreams occurred during the nights patient B demonstrated his nighttime eating behavior.

## Background

### Sleep-related eating disorder

According to the International Classification of Sleep Disorders (ICSD-2; American Academy of Sleep Medicine, 2005), SRED is a non-rapid-eye movement (NREM) sleep parasomnia characterized by recurrent episodes of involuntary compulsive eating during sleep, with morning anorexia and frequent comorbid sleep disorders [e.g., OSA, restless legs syndrome (RLS), sleepwalking; as is the case of our two patients, who both have mild OSA and sleepwalking]. These eating episodes are associated with variable awareness, ranging from dense unawareness, typical of parasomnias like somnambulism, to partial awareness (Winkelman et al., 2011). Morning amnesia for the event is very frequent as well as the undesirable weight gain. SRED appears to combine features of parasomnias (amnesia, automatic behavior, and unresponsiveness to external stimuli) and of an eating disorder (BED; Winkelman et al., 1999). When partially aware of the episode, patients report a strong drive to eat, usually high-caloric foods, whereas excessive exercise and self-induced vomiting, typical of bulimic/anorexic disorders, are missing. SRED can be either primary, or secondary to a medication, like zolpidem (Yun and Ji, 2010), with the two forms being clinically similar. There are no current prevalence data of SRED on the general population. This disorder being heterogeneous (Schenck et al., 1991) the reported prevalence results are inconsistent (0.5–4.7%; Schenck et al., 1991; Winkelman et al., 1999), and they concern groups with different comorbidities (subgroups of psychiatric patients, obese individuals, eating disorder patients, patients with other sleep disorders, and hospitalized patients). This trouble differs from the Night-Eating Disorder (NES), which is a circadian-related nocturnal eating disorder, characterized by bulimic behaviors during a total arousal from sleep, full recall for the event, and the consumption of more than one-third of all daily calories after the evening meal. Obesity and daytime sleepiness are the most important consequences of SRED. Treatment usually concerns the management of the concomitant sleep disorders (RLS and OSA). Dopaminergic agonists are especially effective, especially in patients with RLS (Schenck et al., 1993; Provini et al., 2005). Topiramate, an antiepileptic agent that is efficacious in BED (McElroy et al., 2003), has also shown an efficacy in SRED (Winkelman, 2003, 2006).

The pathophysiology of SRED remains largely unknown. It has been hypothesized that SRED is related to a dysfunctional dopaminergic system (Vetrugno et al., 2006; Provini et al., 2008), mainly due to high prevalence of RLS and the good response to dopaminergic agents in these patients. Furthermore, Khazaal et al. (2003) described a patient treated with the dopaminergic agent bupropion, who developed sleepwalking with overeating behavior. However, no sufficient pathophysiological or functional background has been given to the suggested association of SRED with the dopaminergic system. Recent findings linking compulsive eating to a dopaminergic dysfunction of reward networks, as described in the next section, could offer us supplementary evidence to this hypothesis.

### Dysfunctional reward networks in overeating

Compulsive overeating (e.g., in BED, obesity) has been associated with a dysregulation of DA reuptake (Shinohara et al., 2004) and low striatal D2 receptor availability (Volkow et al., 2008; Johnson and Kenny, 2010). A decrease of D2 and D3 autoreceptors in the midbrain is also possible (Pritchard et al., 2006; Zald et al., 2008). As a compensatory response to reduced receptor availability, DA ML-DA and nigrostriatal dopaminergic activity is accentuated (Bello and Hajnal, 2010; Wang et al., 2011) and prefrontal inhibition is reduced (Volkow et al., 2008), resulting in increased motivation for palatable foods and in compulsive overeating (Wang et al., 2002). This process is similar to compulsive behavior in drug addiction (Koob and Le Moal, 2001; Koob and Volkow, 2010), where the compensatory DA increase in the striatum induces a desensitization of D2 receptors in the ventral striatum, resulting in a tolerance to the effects of a fixed drug dose. When examining personality traits, DA-related traits, such as reward sensitivity, are also related to overeating (Davis et al., 2004). Pharmacological studies have shown that D2 antagonists can potentially reduce food intake (Rusk and Cooper, 1994; Baker et al., 2001), suggesting potential pharmaceutical DA targeting treatments. However, it should be noted that compulsive eating is not explained solely by a dopaminergic dysfunction; other brain networks implicating opioid (Zhang et al., 2003), gamma-aminobutyric acid (GABA; Berridge, 2009), orexin (Piccoli et al., 2012), and *N*-Methyl-d-aspartate (NMDA) transmission (Perogamvros et al., 2012), are also related to eating behavior.

### Active reward processing during sleep

Activation of the ML-DA reward and other instinctual exploratory motivational networks (SEEKING system Panksepp, 1998) is normally present during sleep of mammals (Perogamvros and Schwartz, 2012), and seems to have evolutionary origins (Rial et al., 2010). More specifically, during slow-wave sleep in the rat, reward-related neurons of the ventral striatum are activated (Lansink et al., 2008, 2009). Similarly, during REM sleep reward-related mesolimbic regions are also active including the ventral tegmental area (Dahan et al., 2007), the nucleus accumbens (Lena et al., 2005), and the orexin system (Mileykovskiy et al., 2005), as well as the human hippocampus (with its reward-related theta rhythm Cantero et al., 2003) and the anterior cingulate cortex (Maquet et al., 2000). Besides, sleep deprivation leads to reward dysfunctions like increased risk taking (McKenna et al., 2007), appetitive behavior (Benedict et al., 2012), and overestimation of positive emotional experiences (Gujar et al., 2011). The activation of reward circuits during sleep would be primarily related to memory consolidation, learning enhancement, performance improvement, as well as to the generation of dreams and their motivated content (Perogamvros and Schwartz, 2012). Exploratory and instinctual behaviors in humans are also observed in parasomnias: locomotion in sleepwalking, aggression in REM sleep behavior disorder, sexual behaviors in confusional arousals, and feeding, chewing, or swallowing in the SRED (Vetrugno et al., 2006; Winkelman, 2006). These complex motor behaviors are most often characterized by negative affect (screaming, crying; Oudiette et al., 2009), high motivational value (chewing, swallowing, and sexual behaviors), or even compulsive character (SRED, leg motor activity, sleep-related smoking disorder, and trichotillomania; Murphy et al., 2006; Provini et al., 2008, 2009). Similar characteristics are also found in the normal population (Nielsen et al., 2009). These specific behaviors further support the hypothesis that reward-seeking mechanisms are activated during sleep. Interestingly, most of the reward-related behaviors observed in parasomnias occur during N2 and N3 sleep stages. This may happen because hippocampal ripples, which prevail during these stages (coupled with the sleep spindles Clemens et al., 2007; Clemens et al., 2011), have been implicated in reward processes during sleep (Pennartz et al., 2004; Lansink et al., 2008). In addition, these processes may be more easily expressed in the context of an instable NREM sleep, than REM sleep (Hughes, 2007). Finally, dysregulation of this activation (in insomnia and other sleep disorders) would be implicated in the development of neuropsychiatric disorders, like depression and addiction (Perogamvros and Schwartz, 2012).

## Discussion

Here, we examined two patients with SRED and found that they demonstrate elevated novelty seeking and increased reward sensitivity. In light of new evidence on the ML-DA implication in compulsive eating disorders (see Dysfunctional Reward Networks in Overeating), our findings suggest that the activation of the reward system during sleep (see Active Reward Processing During Sleep) may offer a permissive condition for episodes of nocturnal overeating to occur, especially in patients with elevated reward sensitivity and novelty seeking. However, further research is needed in order to explore the proposed link between reward processing associated personality traits and the expression of SRED.

The screening test for NES confirmed SRED in both patients. Unfortunately, video-polysomnography failed to document a nighttime behavior during the night spent in the laboratory. This is not too surprising given that this behavior does only occur approximately once per week for both patients and may possibly be less frequent in unfamiliar environments like the sleep laboratory. However, in patient A, we found many chewing and swallowing movements during N2 sleep, which have been already described in other SRED patients (Vetrugno et al., 2006). This type of motor behavior may putatively offer a human equivalent for the sniffing reward-anticipating behavior observed in the sleep of rats (Panksepp, 1998; Seelke and Blumberg, 2004). In addition, patient B presented a dissociated arousal in stage N3, which could also suggest vulnerability for parasomniac behaviors.

In the questionnaires assessing reward processing during daytime, the results demonstrate that both patients exhibit increased reward responsiveness, experience seeking, and exploratory excitability, supporting increased ML-DA neurotransmission (see Introduction). These results are similar to the ones found in bulimia nervosa (Battaglia et al., 1996; Rossier et al., 2000) and in overeating behaviors (Davis et al., 2004). Patient A also showed low harm avoidance and behavioral inhibition scores, whereas patient B demonstrated a particularly elevated reward dependence score, suggesting possible modifications in other neurotransmitter systems too (serotonin and norepinephrine). Although only patient A had the criteria for a BED, both patients reported being “voracious” and having a very eager approach to food. A similar increased behavioral approach is also found in the dreams of our patients during the nights they demonstrated their nighttime eating behavior. Elevated exploratory behavior is present in both patients’ dream reports, with the expression of curiosity toward novel stimuli (persons for patient A and situations for patient B).

These findings support the presence of an active reward-seeking network during sleep in humans. Its expression in the form of overt behavior in sleep seems to depend on two conditions, whose concomitancy seems necessary: (a) disinhibition of the central pattern generators of the spinal cord, a condition indispensable for parasomnias (Howell, 2012), and (b) elevated baseline exploratory approach, especially toward a certain kind of stimulus (food in the case of SRED). Further increase in reward-seeking (Puhl et al., 2009; Benedict et al., 2012) after sleep deprivation, which is a main precipitating factor for parasomnias (Joncas et al., 2002), and loss of secondary consciousness during sleep (e.g., self-observation, self-monitoring, planning, decision-making abilities; Hobson, 2009), would also create a facilitating ground for maladaptive seeking behaviors, like compulsive eating. During the parasomniac episode, the posterior cingulate cortex, which was found to be activated in sleepwalking (Bassetti et al., 2000), may be responsible for generating anticipatory biases toward cues (e.g., food) and locations (e.g., kitchen) with elevated motivational value for the subject (Small et al., 2003; Dean and Platt, 2004). In sum, the expression of SRED in our patients would involve motor disinhibition (typical of parasomnias) during sleep together with increased activity within reward regions, due to both the reward system being naturally activated during sleep (section Active Reward Processing During Sleep) and our patients displaying elevated reward sensitivity (assessed by questionnaires). An overt expression of compulsive or other seeking behaviors (chewing, swallowing, and eating) during sleep would be thus unfolded. There also seems to be a facilitatory influence of arousals, as suggested in our patients by the association with mild OSA and the increased arousal index on polysomnography.

## Concluding Remarks

A recent study found that daytime personality traits correlate with the severity of insomnia (negative correlations with novelty seeking, reward dependence, cooperativeness, and positive correlations with harm avoidance, self-transcendence), suggesting a link between individual daytime reward profile and sleep (Park et al., 2012). Our study further investigates this association and, to the best of our knowledge, it is the first to explore the links between daytime personality traits and a particular type of parasomnia. More specifically, the findings presented here suggest plausible physiological links between behaviors expressed during parasomnia episodes and waking personality traits, especially regarding reward-related functions. They thus support the role of an activation of ML-DA reward and other motivational networks (SEEKING system, Panksepp, 1998) during sleep in the expression of SRED. However, our study has some limitations. It examines only two patients with SRED and uses subjective psychometric questionnaires, but not objective experimental measurements, as for example performance on reward tasks. Therefore, future studies with large patient and control samples, and with combined experimental and psychometric testing, as well as brain measurements (EEG/MRI), could help establish these preliminary findings. In particular, investigating other also parasomnias characterized by similar motivational attributes (e.g., sleepwalking, sleep-talking, sleep-driving, bruxism, RBD, nightmares, sleep-related sexual behaviors, and sleep-related smoking syndrome) could contribute to further confirm an active reward processing during human sleep.

## Conflict of Interest Statement

The authors declare that the research was conducted in the absence of any commercial or financial relationships that could be construed as a potential conflict of interest.

## Abbreviations

AASM, American Academy of Sleep Medicine; AHI, apnea–hypopnea index; BAS, behavioral approach system; BED, binge-eating disorder; BES, binge-eating scale; BIS, behavioral inhibition system; BMI, body mass index; BS, boredom susceptibility; C, cooperativeness; DA, dopamine; DIS, disinhibition; EEG, electroencephalogram; EMG, electromyogram; ES, experience seeking; GABA, gamma-aminobutyric acid; HA, harm avoidance; ML-DA, mesolimbic dopaminergic system; MRI, magnetic resonance imaging; N1, N2, N3 stages, stage 1, 2, 3 of NREM sleep; NEQ, Night-Eating Questionnaire; NES, night-eating syndrome; NMDA, *N*-methyl-d-aspartate; NREM sleep, non-rapid-eye movement sleep; NS, novelty seeking; NS1, exploratory excitability subscale of NS; ODI, oxygen desaturation index; OSA, obstructive sleep apnea; P, persistence; PLMs, periodic limb movements during sleep; RBD, REM sleep behavior disorder; RD, reward dependence; RDI, respiratory disturbance index; REM sleep, rapid-eye movement sleep; RERA, respiratory effort-related arousals; RLS, restless leg syndrome; S, self-directedness; SRED, sleep-related eating disorder; SSSV, sensation seeking scale form V; ST, self-transcendence; TAS, thrill and adventure seeking; TCI, Temperament and Character Inventory.
